# Assessment of dental caries predictors in 6-year-old school children - results from 5-year retrospective cohort study

**DOI:** 10.1186/1471-2458-12-989

**Published:** 2012-11-16

**Authors:** Mohd Masood, Norashikin Yusof, Mohamed Ibrahim Abu Hassan, Nasruddin Jaafar

**Affiliations:** 1 Centre of Studies for Community Dentistry, Faculty of Dentistry, Universiti Teknologi MARA, Shah Alam, Malaysia; 2 Centre of Studies for Restorative Dentistry, Faculty of Dentistry, Universiti Teknologi MARA, Shah Alam, Malaysia; 3 Department of Community Dentistry, Faculty of Dentistry, University Malaya, Kuala Lumpur, Malaysia; 4 Community Oral Health Research Group (COHRG), University of Malaya, Kuala Lumpur, Malaysia

## Abstract

**Background:**

This was a retrospective cohort study undertaken to assess the rate and pattern of dental caries development in 6-year-old school children followed-up for a period of 5 years, and to identify baseline risk factors that were associated with 5 years caries experience in Malaysian children.

**Methods:**

This 5-years retrospective cohort study comprised primary school children initially aged 6 years in 2004. Caries experience of each child was recorded annually using World Health Organization criteria. The rates of dental caries were recorded in prevalence and incidence density of carious lesions from baseline to final examination. Risk assessment was done to assess relative risk for caries after 5 years in children with baseline caries status. Simple and multiple logistic regression analysis were performed to identify significant independent risk factors for caries.

**Results:**

The sample consisted of 1830 school children. All components of DMFT showed significant differences between baseline and final examination. Filled teeth (FT) component of the DMFT showed the greatest increases. Results revealed the initial baseline caries level in permanent dentition was a strong predictor for future caries after 5 years (RR=3.78, 95% CI=3.48-4.10, P*<*0.001). Logistic regression analysis showed significant association between caries occurrence and residence (urban/rural) (OR=1.80, *P*<0.001). However, it was not significantly associated with gender and ethnicity. The incidence density of caries, affected persons (ID_p_) observed from baseline and after 5 years was 5.80 persons/100 person-year of observation. The rate of new caries-affected tooth (ID_t_) in the period from baseline and after 5-years was 0.76 teeth/100 teeth-year of observation.

**Conclusion:**

The majority of 12-year-old school children (70%) were caries-free and most of the caries were concentrated in only a small proportion (30%) of them. We found that the presence of caries in permanent teeth at the age of 6 years was a strong predictor of future caries development in this population. The strong evidence of early permanent teeth caries at six years old to predict future caries incidence at 12-year-olds, which could be obtained at almost no cost, questions the need for and cost-effectiveness of expensive technology-based commercial caries predictions kits.

## Background

Dental caries remains a major public health problem due to its widespread nature, high cost of treatment, and it is a main cause of oral pain and tooth loss which ultimately affects quality of life [1,2]. Current evidences show that dental caries is a multifactorial disease and complexly modulated by genetic, behavioral, social and environmental factors [2,3]. Considering this complex etiology of dental caries the major challenge for oral epidemiologists is to identify potential determinants and predictors of dental caries in order to target socially appropriate public health measures to control the disease and its unpleasant consequences [4]. Studies on risk factors of high caries increment have demonstrated that one of the most powerful predictors for dental caries was baseline caries experience in primary teeth. However, others have not found similar results [1,2,5,6].

Apart from identification of predictors, another challenge for oral epidemiologists in prevention of dental caries is its skewed distribution with a certain group of population with high level of caries or at high risk for dental caries development. Although, there has been a significant overall decline in prevalence since the early 1970s worldwide, high disease levels have still been reported in a minority of individuals, labeled as high-caries risk individuals [5]. The distribution of caries level in certain populations is becoming increasingly skewed especially, in developing countries, which leads to the phenomena of disease polarization and increases the inequality in health [7,8]. Over time, a skewed distribution of caries prevalence has developed in many countries, with a minor proportion of 12-year-olds found with high or very high DMFT values [2]. The skewed distribution and high caries risk distribution warrants proper caries risk assessment and prediction in children [6].

Thus, identifying the subpopulation at greatest risk and identification of predictors associated with dental caries prevalence is needed for several reasons [2]. First, knowledge of the main risk indicators and predictors of the disease makes it possible to identify the individuals, who would benefit most from early preventive measures [5]. Secondly, the early identification of these subjects allows health authorities to develop or optimize preventive strategies targeting high-caries risk individuals and to increase the efficiency of preventive programs within the community [7]. A very important consideration for developing countries is the question of cost effectiveness of commercial caries prediction kits which have been marketed indiscriminately to identify those at risk.

The majority of studies investigating risk factors and predictors for dental caries adopted a cross-sectional design or used a cohort design for only a short follow-up period involving relatively small sample size mostly in high income countries [3,4]. Consequently, comprehensive studies of dental caries predictors over a longer duration in a cohort study design involving a population-based sample from low and middle-income countries are needed because the disease trends are contrasting [3,4]. While caries is decreasing in most industrialized countries, the opposite is happening in most developing countries. Therefore, this retrospective cohort study from Malaysia was designed with the objectives to [i] examine the rate and pattern of dental caries development in 6 years old school children followed-up for a period of 5-year, and [ii] to identify baseline risk factors that may be associated after 5 years of caries experience in school children.

## Methods

This was a five-year retrospective cohort study which involved primary school children initially aged 6 years attending government aided schools in the city of Shah Alam and mixed urban–rural districts of Seberang Prai, Malaysia. Both, Shah Alam and Seberang Prai districts have access to fluoridated public water supply at 0.5 to 0.8 ppm since 1976 as part of the national water fluoridation programme. Apart from water fluoridation, fluoridated tooth paste is widely available in both areas [9]. However, topical fluorides are not indicated and rarely used [9].

A significance level of 95% and power of 80% was accepted for the purpose of sample size calculation. Using the proportion-based sample calculation formula [10], a minimum sample size of 737 children was needed for the study. Another sample size calculation was done to test the association of the outcomes with exploratory variables. A sample size of 432 was calculated to detect an odds ratio of at least 1.1 with, significance level of 95% and 80% power, for a caries prevalence of 45% among the non-exposed. To compensate clustering, a design effect of 2 was adopted and the sample size was inflated to double. Moreover, a further 10% was added to compensate possibility of incomplete records and 20% for possible confounding factors in the analysis, totaling 1915 individuals. The initial sample included 1987 six year old children, but 157 records were excluded due to incomplete information throughout the 5 years study period. Therefore, the final sample size consisted of 1830 students.

A multi-stage cluster random sampling was used in the study. At the first stage, all the schools and number of standard six enrolment in each school from both the districts were listed. Calculated sample size (1915) was divided by total number of standard six enrolments in all the schools in both the districts (14035) to calculate probability of selection (0.136) of each student. To maintain equal weight for each student this probability of selection was implemented at each school level and number of sample from each school was calculated. E.g. a sample of 26 students was selected from the school with 192 enrolments (26=192*0.136), whereas, 46 students were selected from the school with 338 enrolments (46=338*0.136).

The research protocol for this study was approved by the Research Ethics Committee of Universiti Teknologi MARA. Parental consent was obtained at the beginning of this programme. Five years data was gathered retrospectively in 2010 from annual school dental records. These record cards included information about demographic data (age, gender and ethnicity), oral health condition (caries experience, gingivitis) and treatment need for each child. All these information in record cards were collected from 2004 to 2009 as part of the School Incremental Dental Care Programme run by Ministry of Health Malaysia. All clinical examination and records were done by trained dental therapists. The clinical data represents records of the dental status and treatment given to each child which was kept by the school administration. Caries experience of each child was recorded annually using World Health Organization (WHO, 1997) diagnostic criteria for caries namely, decayed (D), missing (M), filled (F) teeth (T) and surfaces (S) [10].

The schools were contacted for permission to retrieve the dental records. To maintain confidentiality, the subject’s names were not disclosed. One of the authors (N.Y) examined all the dental records without knowing to which city or school the records belong to. All record cards with complete information from 2004 to 2009 were included for data entry and analysis until the minimum sample size was met. All data were entered by two dental nurses independently to ensure accuracy.

The data were analyzed using the "R-Project" statistical program. The rates of dental caries were presented in prevalence and incidence density of carious lesions from baseline to final examination. Risk assessment was done to assess relative risk for having caries after 5 years in children with baseline caries level. Additionally, bivariate and multivariate logistic regression analysis were performed to establish relationship between dental caries and selected explanatory variables (gender, ethnicity and rural/urban), and odds ratio (OR) and 95% confidence interval (CI) were calculated. Urban and rural classification followed the Malaysian statistics department guidelines. Schools in areas of less than 10,000 inhabitant were classified as rural school and schools in areas of more than 10,000 inhabitants were classified as urban school. In regression analysis, outcome variable was dichotomized according to individuals in the caries free group (DMFT=0, coded as 0) and individuals with caries (DMFT>0, coded as 1). Bivariate logistic regressions were performed individually in separate model with each explanatory variable; model 1 included gender (Female=0 and Male=1), model 2 included ethnicity (Indian=0, Chinese=1, Malay= 3) and model 3 included rural/urban (Rural=0 and Urban=1). Finally, all the explanatory variables were combined into a multivariate logistic regression model.

Incidence in the present study was calculated as incidence density for risk of caries of a person (ID_p_) and of a tooth (ID_t_) summarized by the formula below:

ID_p_= Number of new caries-affected subjects / Total person time at risk for having at least one caries lesion (person -year)

ID_t_= Number of new carious teeth / Total teeth-time at risk (teeth-year)

Person-time in ID_p_ and tooth-time in ID_t_ represent the sum of observation-time for individuals/tooth having no caries. Under School Incremental Dental Care Programme the schools were visited by the examiners at the same time each calendar year for screening and treatment. If a student in a particular school was examined in September 2004, he was followed up in September 2005, 2006, 2008 and 2009. An important assumption for incident density is the accurate recording of the disease-free state of each person at all stages of follow up throughout the duration of study. In case of caries for a person and tooth, it was not clinically feasible to record exact time of onset of caries in a tooth or person. Therefore, we adopted an approach used by Beck et.al. (1997) and Thitasomakul et.al (2006) to calculate caries incidence density in infants [11,12]. It was assumed that the teeth had a uniform risk rate within each follow-up interval. For teeth that were present as sound at baseline (t_0_) and final (t_1_), the time at risk was = t_1_ – t_0_. For teeth which were absent at t_0_, the time of eruption was assumed to be half way between two examinations (t_1_ – t_0_)/2. For teeth that were present as sound at t_0_ and became carious teeth at t_1_, the onset of caries was assumed to take place at halfway between baseline and final examination (t_1_-t_0_)/2. Similarly, if the teeth were absent at t_0_ and became carious teeth at t_1,_ the eruption time was calculated to be at (t_1_ – t_0_)/2, whereas, the onset of caries was assumed to be at t_0_+3/4(t_1_-t_0_). In other words, the caries-free duration of these teeth was one-fourth of the interval. When a carious lesion was detected, that person/tooth was considered to be a new case for the period. It was then excluded from the at-risk status [12].

## Results

A sample of 1830 school children was studied, which comprised of 950 (51.9%) boys and 880 (48.1%) girls. The ethnic mix of the sample consisted of 44.2% Malays, 31.1% Chinese, and 21.5% Indians. About 40.3% of the children were enrolled in Shah Alam schools and 59.7% in Seberang Prai schools. The majority of the children were from urban schools (65.6%) and the rest from rural schools (34.4%). The response rate for this study was 92%. The main reason for dropouts during 5 years was due to absence of student from the school on the day of dental examination. Data on dental caries based on the DMFT index at baseline (aged 6–7 years old) and after 5 years (aged 11–12 years) are presented in Table 1. Total number of teeth, mean DMFT and mean of each component of DMFT at baseline and final examination are shown in Table 2. All components of DMFT showed significant differences between baseline and final examination. Filled (F) teeth component of the DMFT showed the greatest increase as compared to D and M teeth. Table 3 shows the risk assessment analysis of dental caries incidence in subjects who had at least one caries at baseline, and revealed a strong association between the initial baseline condition and that prevailing 5 years later. This was confirmed by results of relative risk tests (RR=3.78, 95% CI=3.48 - 4.10, P*<* 0.001).

**Table 1:** Distribution of children according to the DMFT index at baseline and after 5 years

**DMFT**	**Baseline (at 6–7 years old)**		**After 5 years (at 11–12 years old)**	
	**n**	**%**	**n**	**%**
0	1745	95.4	1281	70.0
1	63	3.4	273	14.9
2	18	1.0	152	8.3
3	3	0.2	73	4.0
4	1	0.1	30	1.6
≥5	0	0	21	1.1
total	1830	100	1830	100

**Table 2:** Components of DMFT index (Decayed, Missing and Filled teeth) at baseline and after 5 years

**Variables**		**Baseline**	**After 5 years**	**P value**^**†**^
Number of teeth				<0.001*
	n	11866	44749	
	Mean	6.49	24.49	
	SD	3.499	3.998	
Decayed teeth				<0.001*
	n	103	238	
	Mean	0.06	0.13	
	SD	0.290	0.459	
Filled Teeth				<0.001*
	n	7	772	
	Mean	0.00	0.42	
	SD	0.091	0.896	
Missing Teeth				<0.001*
	n	0	26	
	Mean	.00	0.01	
	SD	.000	0.132	
DMFT				<0.001*
	n	112	1036	
	Mean	0.06	0.57	
	SD	0.306	1.109	

*Wilcoxon signed ranks test.

^†^Two tailed statistical significance at the 5% level.

**Table 3:** Relative risk assessment for baseline caries as predictor and caries incidence after 5 years as outcome

**Variable**	**Baseline**		**After 5 years**				**P value**^**†**^**and relative risk**
			**Dental caries**		**Caries free**		
	**n**	**%**	**n**	**%**	**n**	**%**	
Dental caries	85	4.6	85	100	-	-	P<0.001
Caries Free	1745	95.4	464	26.6	1281	73.4	RR=3.78
Total	1830	100	549	30.0	1281	70.0	3.48<RR<4.10

^†^Two tailed statistical significance at the 5% level.

Table 4 summarizes the results from bivariate and multivariate logistic regression analysis in relation to categories of independent variables. Caries occurrence was associated with location of residence (*P*< 0.001), however, it was not significantly associated with gender and ethnicity. Table 4 also shows the crude and adjusted odds ratios after multivariate analysis. Crude odds ratio represent how much greater the probability of disease among students affected by a risk factor. Since, a hidden effect of a confounder variable may present, the independent effect of each variable is also presented (adjusted odds ratio). Compared to the Indian ethnic group students the odds of caries was 1.04 times higher for Malay and 1.22 times higher for Chinese students, in the crude models. These odds decreased slightly in the adjusted model to 0.99 and 1.12 among the Malay and Chinese students, respectively. In the crude model, children who lived in urban areas showed 80% greater probability of presenting caries when compared to those children residing rural areas. After adjusting, this differences dropped to 75%, but remained statistically significant. In relation to gender, the crude model revealed greater odds of dental caries in males (1.19; 95% CI= 0.97-1.46). However, the association of this variable decreased (1.16; 95% CI= 0.95-1.41) after adjustment for the confounding effect of location and ethnicity.

**Table 4:** Bivariate and Multivariate logistic regression model for independent variables' effect on caries increment at baseline and after five years

**Independent Variable**		**n(%)**	**Caries free after 5 years n(%)**	**Caries free p**^**†**^**Value**	**Crude**			**Adjusted**		
					**OR**	**95% CI**^**††**^	**P**^**†**^	**OR**	**95% CI**^**††**^	**P**^**†**^
Gender				0.153			0.088			0.143
	Male	950 (51.1)	679 (71.4)		1.19	0.97-1.46		1.16*	0.95-1.41	
	Female	850 (48.1)	602 (68.4)		1			1		
Ethnicity				0.558			0.122			0.958
	Malay	808 (44.2)	561 (69.4)		1.04	0.82-1.32		0.99**	0.78-1.25	
	Chinese	570 (31.1)	395 (69.3)		1.22	0.94-1.58		1.12**	0.87-1.45	
	Indian	449 (24.5)	323 (71.9)		1			1		
Urban/Rural				<0.001			<0.001			<0.001
	Urban	1200 (65.6)	890 (74.2)		1.80	1.46-2.22		1.75***	1.42-2.1	
	Rural	630 (34.4)	391 (62.1)		1			1		

Odds ratio adjusted for ethnicity and urban/rural (*), gender and urban/rural (**), gender and ethnicity (***).

^†^Two tailed statistical significance set at 5% level.

^††^ Confidence Interval.

Table 5 shows incidence density rate for caries affected teeth and children. The number of caries-affected person and teeth were 1038 and 464, respectively. The incidence of caries, affected persons (ID_p_) observed from baseline and after 5 years was 5.80 persons/100 person-year of observation. The rate of new caries-affected tooth (ID_t_) in the period from baseline and after 5 years was 0.76 teeth/100 teeth-year of observation.

**Table 5:** Frequency, incidence and incidence density ratio of caries affected children and teeth

	**Observation period (5 Years)**
n(Children)	1830
Number of new caries affected cases	464
Children-years of observation	7990
ID_p_/100 children at risk	5.80
n(teeth)	44829
Number of new caries affected teeth	1038
Teeth-years of observation	137099.7
ID_t_/100 teeth at risk	0.76

ID_p_ Incidence density for person.

ID_t_ Incidence density for teeth.

## Discussion

The present study provided interesting information on the current dental caries status, increment and associated factors (previous caries experience, ethnicity and urbanization) in a five year retrospective cohort study involving a relatively large sample size of 1830 school children in Malaysia. The prevalence and severity of dental caries among Malaysian school children was already quite low as reported by several studies and national surveys [13-15]. The present study showed that the national target for oral health of 12-year-olds by the year 2010 [16,17], i.e. at least 60% children are caries-free and mean DMFT of less than 1.5 had been achieved with more than 70% caries-free children and a very low mean DMFT of 0.58.

Although, the overall caries level is very low in the study population, but the worry was the markedly skewed distribution of dental caries with the majority of the disease concentrated in a minority of the population i.e. in 6% students in 6-year-olds and 30% in 12-year-olds. These findings indicate that there were inequalities and the skewed distribution existed at various levels in dental caries distribution in Malaysian school children. The identification of such a high risk group early in life may allow tailored interventions for individual children including diet and nutrition counseling, behavioral approach and other appropriate preventive measures such as targeted topical fluoride applications [18]. We propose a policy of screening examination such as a compulsory dental health checkup for 6 year olds coinciding with the age of the first permanent tooth eruption to identify children at risk (i.e. any child with caries experience) followed immediately by intensive caries preventive measures for this high risk group. This might be a worthwhile and more cost-effective and cost-efficient public health approach [7] as compared to using costly and time consuming commercial caries risk prediction test kits such as salivary tests and lactobacillus counts. The present caries trends support the monitoring of localized dental health strategies and facilitate the targeting of resources to help reduce the current inequalities in dental health among school children in Malaysia [8].

Due to the skewed distribution of dental caries, the importance of identifying caries predictors in permanent dentition was emphasized in this study. Risk indicators and predictors of caries in children have been investigated in many cross-sectional studies as well as a few longitudinal studies. They showed that past caries experience in the primary dentition was a powerful predictor of future disease [5,18]. Nevertheless, the relationship between caries experience in young permanent dentition to predict new caries development of future permanent teeth has not been sufficiently established [18]. Predicting future caries is important for monitoring individuals at risk of developing caries [6]. The most important factor for future dental health is the preservation of healthy permanent first molars, which erupt first and are usually present in 6-year old children. It is the most caries susceptible teeth in the young permanent dentition. Thus, the risk factor for later caries should be assessed at the age of 6 years when the first permanent tooth erupts in the oral cavity and its progress should be followed annually for a certain period of time [1,2].

This study showed that there was an association between caries experience in young permanent teeth at 6-year-olds with new caries increment in later life. Children with caries in the permanent teeth at 6-year-olds were nearly four times more likely to experience new caries over the 5-year period. In other words, no caries experience at baseline was a good predictive factor against high caries increment [6].

Caries experience is associated with urbanization and ethnicity in Malaysia, where a few studies including the national oral health surveys have consistently reported these trends [7]. The present study found the association between urbanization and prevalence of caries in the multiple regression analysis (Tables 3 and 4). Urbanization at baseline was an important factor associated with caries development during the succeeding 5-year period, and the adjusted and unadjusted odds ratio for urbanization was significantly high [18].

This study also analysed the caries increment in terms of incidence density. There were several reasons to include the method of incident density in this study. First, a cohort study is suitable for the calculation of an incidence rate and better characterizes the incremental rate of disease events. Secondly, this more precisely estimates the rate of disease occurrences as it accounts for the varying times of follow up where the prevalence and incidence rates do not [12]. Third, this is of importance as the length of follow up was not consistent for all teeth. Some teeth erupted and some entered the study later than others [19]. To reduce the high caries rate among the high-risk population, a preventive program must be implemented early in life at the time of tooth eruption.

The strength of the present study was the retrospective assessment of a cohort of 1830 school children for 5 years. However there were some limitations. The first limitation of this study was that it relied on uncalibrated dental caries assessment, which were recorded by different dental therapists annually over the five-year period, this may impact the internal validity of the collected data. However, although the therapists were not calibrated, they received similar professional training and used standard WHO (1997) criteria for screening and recording caries. In turn, these data were used for the actual treatment and updated annually in the Incremental Dental Care system. During the 5 years duration of this study, each participant was examined every year by different examiner on the same dental record card. During the examination, the current year dental charting was matched with previous years dental charting records. We are of the opinion that this procedure of repeated measure and matching of dental charting records every year improved the accuracy of the record. We advocate the use of annual treatment records as a means of monitoring disease trends because it is readily available, cheap and timely, as compared to purposive epidemiological surveys. We believe that data which is timely is better for monitoring than accurate data which may be outdated by the time the data is analyzed and published. In addition, data from records do provide a very useful tool to monitor disease trends because they do not incur additional costs or the need to organize costly nationwide surveys at 5 or 10 year intervals.

Secondly, we did not analyze all possible confounding effects of other biological, socioeconomic and environmental variables commonly associated with dental caries experience in this age range (such as salivary mutans streptococci, saliva pH or salivary flow etc.) which was beyond the normal scope of the national school incremental dental care programme besides being prohibitively expensive and unjustifiable [7]. Thus, the present study used urban / rural school category as the nearest proxy to reflect socio-economic status. A previous national survey supports this assumption because there was a clear inverse association of caries with parental education level and monthly household income [15]. As for the possible confounding effect of pattern of dental visit, this was not considered as an important confounding factor in Malaysia because the school dental service provides free dental examination and treatment for all school children. The present coverage of schools nationwide is over 95%, while all schools involved in the present study were covered.

## Conclusion

In conclusion, we found that the majority (70%) of 12-year-old school children in the sample were caries free. However, the majority of caries were concentrated in a small proportion (30%) of school children. The study found that the presence of caries in the permanent teeth at the age of 6 years was a strong predictor of future caries development in this population. Moreover we felt that the strong evidence of early permanent teeth caries at six years old to predict future caries incidence which could be obtained at almost no cost, far outweighs the cost-benefit and cost-efficiency value of expensive technologically complex commercial caries predictive kits (such as salivary tests, bacteriological counts etc.) which may not be reliable in the long term.

## Competing interests

The author(s) declared no potential conflicts of interest with respect to the research, authorship, and/or publication of this article.

## Authors' contributions

All the authors provided input into drafts and approved the final draft of the manuscript. MM contributed to data analysis and manuscript writing; NY contributed to the design of the study and write up. MIAH and NJ contributed to conceptual framework of the project and data interpretation. All authors read and approved the final version of the manuscript.

## Pre-publication history

The pre-publication history for this paper can be accessed here:

http://www.biomedcentral.com/1471-2458/12/989/prepub
